# Consolidated bioprocessing of butanol production from xylan by a thermophilic and butanologenic *Thermoanaerobacterium* sp. M5

**DOI:** 10.1186/s13068-018-1092-1

**Published:** 2018-04-02

**Authors:** Yujia Jiang, Dong Guo, Jiasheng Lu, Peter Dürre, Weiliang Dong, Wei Yan, Wenming Zhang, Jiangfeng Ma, Min Jiang, Fengxue Xin

**Affiliations:** 1 State Key Laboratory of Materials-Oriented Chemical Engineering, College of Biotechnology and Pharmaceutical Engineering, Nanjing Tech University, Puzhu South Road 30#, Nanjing, 211800 People’s Republic of China; 2 Jiangsu National Synergetic Innovation Center for Advanced Materials (SICAM), Nanjing Tech University, Nanjing, 211800 People’s Republic of China; 3 Institute of Microbiology and Biotechnology, University of Ulm, 89069 Ulm, Germany

**Keywords:** *n*-Butanol, Xylan, Bifunctional alcohol/aldehyde dehydrogenase (AdhE), *Thermoanaerobacterium*, Co-cultivation

## Abstract

**Background:**

Consolidated bioprocessing (CBP) has attracted increasing attention since it can accomplish hydrolytic enzymes production, lignocellulose degradation and microbial fermentation in one single step. Currently, biobutanol is mainly produced by mesophilic and solventogenic clostridia, such as *Clostridium beijerinckii* and *C. acetobutylicum*, which cannot directly utilize lignocellulose, an abundant, renewable and economic feedstock. Hence, metabolic construction or isolation of novel cellulolytic/hemicellulolytic and solventogenic bacteria to achieve direct butanol production from lignocellulose offers a promising alternative.

**Results:**

In this study, a newly isolated *Thermoanaerobacterium* sp. M5 could directly produce butanol from xylan through CBP at 55 °C via the butanol–ethanol pathway. Further genomic and proteomic analysis showed that the capabilities of efficient xylan degradation and butanol synthesis were attributed to the efficient expression of xylanase, β-xylosidase and the bifunctional alcohol/aldehyde dehydrogenase (AdhE). Process optimization based on the characteristic of AdhE could further improve the final butanol titer to 1.17 g/L from xylan through CBP. Furthermore, a new co-cultivation system consisting of *Thermoanaerobacterium* sp. M5 which could release xylose from xylan efficiently and *C. acetobutylicum* NJ4 which possesses the capacity of high butanol production was established. This microbial co-cultivation system could improve the butanol titer to 8.34 g/L, representing the highest butanol titer from xylan through CBP.

**Conclusions:**

A newly thermophilic and butanogenic bacterium *Thermoanaerobacterium* sp. M5 was isolated and key enzymes responsible for butanol production were characterized in this study. High butanol titer was obtained from xylan through process optimization. In addition, the newly set up microbial co-cultivation system, consisting of *Thermoanaerobacterium* sp. M5 and *C. acetobutylicum* NJ4, achieved the highest butanol production from xylan compared with the reported co-cultivation systems.

## Background

Butanol, a four-carbon and straight-chained alcohol, is considered as a more advanced biofuel over ethanol, owing to its higher heating value [1], better intersolubility [2], lower heat of vaporization [3], higher viscosity and lower corrosivity [4]. Nowadays, biobutanol is mainly produced through traditional acetone–butanol–ethanol (ABE) fermentation process with a typical mass ratio of 3:6:1 conducted by several mesophilic solventogenic clostridia, such as *Clostridium acetobutylicum*, *C. beijerinckii*, *C. saccharobutylicum* and *C. saccharoperbutylacetonicum* [5, 6]. The economics of ABE fermentation is greatly affected by costly traditional feedstocks, such as starchy based materials and molasses. Lignocelluloses, such as wheat straw and sugarcane bagasse, are ideal substrates owing to their abundance, renewability and non-food competition with human demand; however, costly pretreatment and hydrolysis techniques are required as solventogenic clostridia cannot directly utilize polysaccharides, such as cellulose/hemicellulose [7, 8].

Biofuel generation from cellulose/hemicellulose without supplementation of exogenous cellulases/xylanases, known as consolidated bioprocessing (CBP), is believed to reduce costs substantially compared to the process in which cellulose/hemicellulose degradation and microbial fermentation are accomplished in separate steps [9]. Although several cellulolytic/hemicellulolytic *Clostridium* sp. can produce ethanol from lignocellulose through CBP, such as *C. cellulolyticum* and *C. thermocellum*, no wild-type cellulolytic/hemicellulolytic *Clostridium* sp. can indigenously synthesize butanol directly from lignocellulose due to the lack of butanol synthetic pathway. Only through the integration of the butanol synthetic pathway into these cellulolytic/hemicellulolytic *Clostridium* sp., butanol production from cellulose/hemicellulose via CBP can been achieved. For instance, 0.12 g/L of butanol was produced from crystalline cellulose after introduction of the CoA-dependent pathway by the recombinant *C. cellulolyticum* after 20 days. In addition, it should be noticed that the butanol titer by the recombinant was still maintained at low levels due to the low cellulolytic degradation rate under mesophilic conditions [10].

Compared to the mesophilic process, thermophilic processes show greater prospects in achieving CBP owing to its relatively high degradation efficiency [11, 12]. In addition, thermophilic conditions could avoid microbial contamination, decrease the cooling costs and further facilitate the downstream product recovery [13]. Currently, most wild-type thermophilic strains, such as *C. thermocellum*, *Thermoanaerobacterium saccharolyticum* and *Geobacillus thermoglucosidasius*, have been widely adopted for improvement of ethanol production through genetic modification. Among these thermophilic strains, *C. thermocellum* is the most well studied and shows efficient cellulose degradation; however, it cannot utilize hemicellulose, which is the second most abundant natural resource. *T. saccharolyticum* is another commonly used thermophilic species; however, it lacks the capability to produce butanol [14]. Through introduction of the butanol synthetic pathway in a lactate-deficient *T. saccharolyticum* strain, 1.05 g/L of butanol was finally produced from 10 g/L xylose [12]. However, butanol production from cellulose/hemicellulose by *T. saccharolyticum* has not been reported [12]. Considering the abundance of cellulose/hemicellulose and low level of butanol production, novel thermophilic strains or processes are still urgently needed to achieve high butanol production from cellulose/hemicellulose through CBP.

In this study, a thermophilic and butanogenic *Thermoanaerobacterium* sp. M5 was isolated and identified, which exhibited relatively high capacity of xylan degradation and butanol production through a butanol–ethanol (BE) fermentation pathway instead of the traditional ABE one. Key enzymes responsible for xylan degradation and BE formation were also studied. Finally, a microbial co-cultivation system was set up to further improve final butanol production from xylan through CBP.

## Methods

### Microorganism and growth media

The bacterium was isolated from the decomposite soil collected from Laoshan Nature Park, China, and cultivated in a reduced mineral salts medium, which contained 1.0 g/L NaCl, 0.5 g/L MgCl_2_·6H_2_O, 0.2 g/L KH_2_PO_4_, 0.3 g/L NH_4_Cl, 0.3 g/L KCl and 0.015 g/L CaCl_2_·2H_2_O. The media were also supplemented with 1 mL trace element solution, 1 mL Na_2_SeO_3_–Na_2_WO_4_ solution and 3 g yeast extract in 1 L of the medium [15]. The trace element solution contains: (g/L) FeCl_2_·4H_2_O 1.5, CoCl_2_·6H_2_O 0.19, MnCl_2_·4H_2_O 0.1, ZnCl_2_ 0.07, Na_2_MoO_4_·2H_2_O 0.036, NiCl_2_·6H_2_O 0.024, H_3_BO_3_ 0.006, CuCl_2_·2H_2_O 0.002. The Na_2_SeO_3_–Na_2_WO_4_ solution contains: (g/L) Na_2_SeO_3_·5H_2_O 0.006 and Na_2_WO_4_·2H_2_O 0.008. Beechwood xylan purchased from Sigma-Aldrich Co. Ltd. (Shanghai, China) was used as the carbon source. In addition, 20 mM *N*-[Tris (hydroxymethyl) metyl]-2-aminopropanesulfonic acid (TES) and 10 mg resazurin were added as the pH buffer agents and oxygen indicator, respectively. 0.2 mM Na_2_S, 0.2 mM l-cysteine and 0.5 mM dl-dithiothretol were finally added as reductants. 40 mL medium was divided in 100 mL serum bottles purged with N_2_ (nitrogen), then autoclaved for 15 min and cooled down to room temperature. After six transfers in medium with xylan as the sole carbon source, the enriched culture was diluted 10^5^ times and screened on agar plates with the Congo red staining method. The bacteria were inoculated by a single colony from an agar plate. *C. acetobutylicum* NJ4 was pre-cultured for 60 h when OD reached 4.0–5.0. Then, strain NJ4 was inoculated into the culturing medium of strain M5 after 72 h. The fermentation can be divided into two stages. The first is the xylan degradation stage (0–72 h), in which the temperature and pH were maintained at 55 °C and 7.5; the following one is the butanol production stage (72–192 h), in which the temperature and pH were 37 °C and 6.0. For maintenance of the pH, various buffers with different buffering capabilities were used in this study: citrate buffer, pH 4.0–6.0; PBS, pH 6.0–8.0; and glycine–NaOH buffer, pH 8.0–10.0. In addition, pH was adjusted every 12 h regularly. The sampling intervals were 24 h and the data were obtained for at least triplicates. The processes of enrichment, isolation and cultivation were all operated in an anaerobic chamber filled with N_2_ and the culture temperature maintained at 55 °C. The strain M5 was preserved in China Center for Type Culture Collection (Wuhan, China) under the deposit number of CCTCC M2017072.

### Identification of bacterial isolates using 16S rDNA gene sequences

The genomic DNA of cell pellets from isolate M5 was extracted and purified with the genomic DNA of M5 as the template, and PCR amplification of the 16S rDNA genes was performed with a pair of universal bacterial primer 27 forward primer: 5′-AGAGTTTGATCC TGGCTCAG-3′, and 1492 reverse primer: 5′-TACGGCTACCTTGTTACGACTT-3′. Then the purified PCR products were sequenced and compared to available databases using the Basic Local Alignment Search Tool (BLAST) to determine the approximate phylogeny. The nucleotide sequence of culture M5 was deposited in the GenBank under an Accession Number of MF405082.1.

### The draft complete genome sequencing and analysis of strain M5

The bacterium was cultivated in mineral salts medium amended with xylose as the sole carbon source for 3 days. The genomic DNA was extracted using G^+^ bacteria genomic DNA kit (ZOMANBIO, China). The large fragment of genomic DNA was firstly broken into small sequences with an average 300 bp by Covaris M220 (Covaris, USA), and the DNA libraries were constructed via NEBNext^®^ UltraTM DNA Library Prep Kit for Illumina (NEB, USA). Then the draft genome was sequenced using Illumina HiSeq2500 system sequencing technology. Due to the raw reads containing spike-in and low-quality reads, the raw reads should be trimmed and filtered to obtain the clean reads for the high quality of data analysis via the Trimmomatic v0.32. Finally, filtered reads were assemble de novo through the method of de Bruijn graphs via velvet v1.2.03, and all open reading frames (ORFs) were predicted by Glimmer3 v3.02 due to the flexibility of the algorithm. Lastly, the genes were annotated through the Nucleotide collection (Nr/Nt) of NCBI and Kyoto Encyclopedia of Genes And Genomes (KEGG) databases, and constructed into metabolic pathways through KEGG.

### Enzymatic assays

The activity of xylanase was determined by the 3,5-dinitrosalicylic acid (DNS) method. The 1 mL of culture supernatant was added in 1 mL PBS (50 mM pH 6.5) containing 1% (w/v) xylan and incubated at 55 °C for 10 min. 2 mL DNS was added to terminate the reaction and detect the content of reducing sugars. Finally, the amount of reducing sugars was calculated by measuring the increasing absorbance at 540 nm according to the xylose standard curve. One unit (U) of xylanase was defined as the amount of enzyme that was able to release 1 μmol xylose per min from xylan.

β-Xylosidase activity depends on the amount of *p*-nitrophenyl (*p*NP) released from *p*-nitrophenyl-β-d-xylopyranoside (*p*NPX). The reaction mixture contained 1 mL of enzyme solution and 1 mL 8 mM *p*NPX in 50 mM, pH 6.5 PBS. Then the reaction was incubated at 55 °C for 10 min and stopped by the addition of 1 mL 1 M Na_2_CO_3_ and then the increasing absorbance at 405 nm measured. One unit (U) of β-xylosidase activity was defined as the amount of enzyme that releases 1 μmol of *p*NP per min from the synthetic substrate *p*NPX.

The activity of butanol dehydrogenase (BDH) was measured by monitoring the NADH consumption at 340 nm. 15 mM of butyraldehyde and 0.3 mM NADH was added together in 2 mL PBS (50 mM, pH 6.0). Then, 1 mL cell lysate was added and the reaction initiated. The NADH–BDH activity was assayed by monitoring the reducing absorbance at 340 nm, and the amount of reduced NADH was calculated through its standard curve. One unit (U) of BDH was defined as the amount of enzyme which consumed 1 μmol NADH per minute.

### Characterization of key proteins by LC–MS/MS analysis

The cells were removed by centrifugation at 12,000*g* at 4 °C for 10 min. The protein solution was reduced with 5 mM dithiothreitol (DTT) for 30 min at 56 °C and alkylated with 11 mM iodoacetamide (IAA) for 15 min at room temperature in darkness. Finally, trypsin was added at 1:50 trypsin-to-protein mass ratio for the first digestion overnight and 1:100 trypsin-to-protein mass ratio for a second 4 h digestion. The tryptic peptides were fractionated into fractions by high pH reverse-phase column (Agilent 300Extend C_18_; 5 μm particles, 4.6 mm ID, 250 mm length). Briefly, peptides were first separated with a gradient of 8–32% acetonitrile (pH 9.0) over 60 min into 60 fractions. Then, the peptides were combined into four fractions and dried by vacuum centrifuging. The tryptic peptides were dissolved in 0.1% formic acid (v/v, solvent A) and directly loaded onto a homemade reversed-phase analytical column (15-cm length, 75 μm i.d.). The gradient comprised an increase from 6 to 25% solvent B (0.1% formic acid in 90% acetonitrile) for 24 min, and ramped to 25–40% solvent B in 8 min, then climbing to 80% solvent B for 4 min and lastly holding at 80% solvent B for 4 min. The flow rate was maintained at 380 nL/min on an EASY-nLC 1000 UPLC system. The resolved peptides were subjected to NSI source followed by tandem mass spectrometry (MS/MS) in Q Exactive™ Plus (Thermo) coupled online to the UPLC. The resulting MS/MS data were processed using Maxquant search engine (v1.5.2.8).

### Cloning, expression and purification of a bifunctional alcohol/aldehyde dehydrogenase (AdhE)

The *adhE* gene from strain M5 was amplified using the primers F/R (F: 5′-TAAGAAGGAGATATACCATGGGCCAAATAGACGCAATAGTAAAGGCAATGGC-3′; R: 5′-GTGGTGGTGGTGGTGCTCGAGTGCACCGTATGCTTTTCTGTAGATCTC-3′) and PrimeSTAR^®^ HS DNA Polymerase (TaKaRa, Shanghai, China). The PCR conditions were: denatured at 95 °C for 10 min, followed by 30 cycles of 98 °C for 10 s, 55 °C for 15 s, and 72 °C for 2 min 40 s, then 72 °C for 10 min. PCR fragment and plasmid of pET28a (+) were digested using *Xho* I and *Nco* I and ligated using One Step Cloning Kit (Vazyme Biotech. Co., Ltd., Nanjing, China). The plasmids harboring *adhE* gene fragment were transformed into *E. coli* BL21 (DE3), which was incubated in LB medium with 30 μg/mL of kanamycin at 37 °C for 12 h. 0.05 mM isopropyl-β-d-thiogalactopyranoside (IPTG) was added and the culture temperature was lowered to 18 °C for 20 h. The recombinant protein with His_6_-tags was purified with Ni^2+^–NTA resin (Qiagen, Valencia, CA, USA). After elution of non-target proteins with 25 mM imidazole in 20 mM PBS (pH 7.0), the target fusion protein was eluted with a linear concentration gradient of imidazole in 20 mM PBS (pH 6.0). The proteins were identified using sodium dodecyl sulfate polyacrylamide gel electrophoresis (SDS-PAGE) and visualized by staining with Coomassie Brilliant Blue R-250. SDS-PAGE was performed on a 12% gel using electrophoresis apparatus at 80 V for the first 60 min, followed by 120 V for 2 h.

The optimal reaction pH was assessed at 55 °C using the following buffers: 20 mM citrate buffer, pH 4.0–6.0; 20 mM PBS, pH 6.0–8.0; and 20 mM glycine–NaOH buffer, pH 8.0–10.0. The effect of the temperature on BDH activity was determined under the optimal pH at temperatures ranging from 35 to 85 °C. To measure the pH stability, the enzyme was incubated at 4 °C for 1 h in different buffers and the residual activity was determined using the enzyme assay conditions described above. The thermal stability of BDH was assessed by incubating the enzyme preparations at different temperatures for a certain time until the remaining activity decreased below 30% of its initial activity. Non-heated enzyme was used as the control (100%).

### Analytic methods

Concentrations of xylose were quantified by high-performance liquid chromatography (HPLC) (UitiMate 3000 HPLC system, Dionex, USA) at a wavelength of 215 nm on a UVD 170U ultraviolet detector, and an ion exchange chromatographic column (Bio Rad Aminex HPX-87H column, USA) was used. The products were eluted at 55 °C with 5 mM H_2_SO_4_ as the mobile phase at a flow rate of 0.6 mL/min. Metabolic products, such as ethanol, acetate, butanol and butyrate, ware detected by gas chromatography (GC-2010, Shimadzu Scientific Instruments, Japan) equipped with an InterCap WAX column (0.25 mm × 30 m, GL Sciences Inc., Japan) and a flame ionization detector (FID). The column temperature was held at 60 °C for 2 min and increased to 150 °C at a rate of 30 °C/min. The temperature of the injector and detector was both set at 180 °C. Nitrogen was used as the carrier gas, with a flow rate of 30 mL/min. All samples were centrifuged at 12,000*g* for 5 min at 4 °C, then 50 μL HCl (2 M) was added in 950 μL of the samples. Isobutanol was used as the internal standard.

## Results

### Isolation and phylogenetic identification of a thermophilic *Thermoanaerobacterium* sp. M5 capable of synthesizing butanol through the BE pathway

An anaerobic colony named M5 with relatively high activity of xylanase was identified on agar plates using beechwood xylan as the sole carbon source at 55 °C. Further fermentation without pH adjustment using mineral salts medium spiked with 20 g/L of beechwood xylan as the sole carbon source was carried out for 5 days. After comprehensive detection with GC-FID and HPLC, the final metabolic products contained 0.76 g/L of ethanol, 0.23 g/L of butanol, 3.49 g/L of acetate and 4.84 g/L of butyrate, indicating that strain M5 could directly synthesize butanol from xylan through CBP. Meanwhile, the elimination of acetone suggests that strain M5 may possess a BE pathway. Further phylogenetic analysis of the 16S rDNA gene (NCBI Accession Number MF405082.1) showed that strain M5 belonged to *Thermoanaerobacterium* sp. with 99% similarity with *T. thermosaccharolyticum* KKU19 (NCBI Accession Number JN020648.1) (Table 1).

**Table 1:** Top matches of the 16S rDNA sequence of strain M5 against known bacterial sequences from the Genbank database (BLAST, NCBI)

Bacterium	Accession	Query cover (%)	Identify (%)
*T. thermosaccharolyticum* strain JCA-5637 16S ribosomal RNA, partial sequence	LC127099.1	99	99
*T. thermosaccharolyticum* strain KKU19 16S ribosomal RNA gene, partial sequence	JN020648.1	99	99
*T. thermostercoris* strain Buff 16S ribosomal RNA gene, partial sequence	NR_122103.1	98	99
*Thermoanaerobacterium* sp. K162C 16S ribosomal RNA gene, partial sequence	HQ840649.2	99	98
*T. thermosaccharolyticum* M0795, complete genome	CP003066.1	99	98
*T. thermosaccharolyticum* strain DSM 571 16S ribosomal RNA, partial sequence	NR_074419.1	99	98
*Thermoanaerobacterium* sp. M5 (this study)	MF405082.1	100	100

*Thermoanaerobacterium* sp. has been reported to be a group of endospores, forming motile, rod-shaped and obligate anaerobic prokaryotes, which are broadly used for hydrogen production [16]. However, few studies regarding butanol production directly from xylan by thermophilic *T. thermosaccharolyticum* have been reported. Therefore, this newly isolated wild-type butanogenic *Thermoanaerobacterium* sp. M5 further broadens our knowledge and adds to the pool of known butanol-generating microbes.

### Genomic sequencing, annotation and comparison of strain M5 with other thermophilic and solventogenic strains

To better understand the genomic information and elaborate the xylan degradation and butanol synthetic pathways, the whole genome of *Thermoanaerobacterium* sp. M5 was sequenced and annotated. The genomic sequence of strain M5 was deposited under the GenBank Accession No. of NDHF00000000. The final assembly of the genome generates 79 contigs ranging from 525 to 225947 bases with 33.9% G+C content, 7 rRNAs and 55 tRNAs. Strain M5 consists of an approximate 2.64 Mbp chromosome, which is lower than those of other thermophilic sp., such as 3.84 Mbp of *C. thermocellum* and 3.87 Mbp of *G. thermoglucosidasius*. The 2.64 Mbp chromosome of strain M5 is only slightly lower than 2.79 Mbp of *T. thermosaccharolyticum* DSM 571 and 2.73 Mbp of *T. saccharolyticum*, and higher than 2.31 Mbp of *T. mathranii*. However, it possesses more coding sequences (CDS, 2622) than those of *T. thermosaccharolyticum* DSM 571 (2568) and *T. mathranii* (2152), and similar to that of *T. saccharolyticum* (2643).

### LC–MS/MS analysis of key enzymes involved in xylan degradation within *Thermoanaerobacterium* sp. M5

Although solventogenic *Clostridium* sp. contains some polysaccharides degrading genes, such as cellulase and xylanase, they cannot directly utilize lignocellulose due to the expression of these enzymes [17]. Currently, *T. saccharolyticum* was the most efficient xylan degrader with ethanol as the metabolic product [14]. In this study, the newly isolated *Thermoanaerobacterium* sp. M5 cannot only efficiently degrade up to 90 g/L of xylan, but also produce both butanol and ethanol as final metabolic products. Furthermore, strain M5 could efficiently secrete xylan-degrading enzymes, such as xylanase and β-xylosidase to hydrolyze xylan. For example, when 30 g/L of xylan was adopted as the sole carbon source, strain M5 could secrete 0.26 U/mg of xylanase and 3.81 U/mg of β-xylosidase in the fermentation broth after 48 h, suggesting that strain M5 could efficiently secrete xylan-degrading enzymes.

To further identify xylan hydrolytic enzymes, proteins in the fermentation supernatant using xylan as the sole carbon source were collected. After being subjected to trypsin digestion, the samples were analyzed by LC–MS/MS. Based on the resulting MS/MS data evaluated by the Uniprot *T. thermosaccharolyticum* database, glycoside hydrolases, mainly xylanase, xylosidase, glucuronidase and alpha-galactosidase were identified (Table 2). Among these proteins, one xylanase (D9TMZ9) and two β-xylosidases (D9TMZ0 and D9TMY9) were annotated, which are synergistically responsible for xylan degradation [18]. The domain architecture of D9TMZ9 consists of CBM4-GH10-CBM9-CBM9-SLH-SLH-SLH, which possesses 1288 amino acids with 141.5 kDa molecular masses. According to the unique peptides, D9TMZ9 contains three main domains: carbohydrate-binding modules (CBMs), GH10 domain and the SLH (for S-layer homology) domain. The GH10 domain plays a vital role in the degradation of xylan, which implements the random hydrolysis of internal β-(1,4)-xylosidic linkages in the insoluble xylan backbone to soluble xylooligosaccharides [19]. The GH10 domain in D9TMZ9 possesses 276 amino acids with the predicted 31.4 kDa molecule mass and a (β/α)_8_-fold structure. In addition, the CBMs are distinct in their selectivity for binding amorphous lignocellulose, and SLHs are paracrystalline mono-layered assemblies of (glyco)proteins, which coat the surface of bacteria with about 50–60 residues. These two domains facilitate the combination with xylan and promote xylan degradation. The two identified xylosidases, D9TMZ0 and D9TMY9, belong to GH39 and GH52, respectively, with the predicted molecular weights of 58.5 and 77.0 kDa, which could further hydrolyze the terminal xylose monomers from the non-reducing end of xylooligosaccharides.

**Table 2:** Secreted proteins of *Thermoanaerobacterium* sp. M5 identified by LC–MS/MS

Protein accession	Description	Gene name	MW [kDa]	#Unique peptides
D9TMZ9	Beta-xylanase	Tthe_0992	141.45	12
D9TMZ0	Xylan 1,4-beta-xylosidase	Tthe_0983	58.468	1
D9TMY9	Xylan 1,4-beta-xylosidase	Tthe_0982	76.957	7
D9TMY6	Xylan alpha-1,2-glucuronidase	Tthe_0979	78.844	12
D9TLK2	Glucan 1,4-alpha-glucosidase	Tthe_0773	78.399	12
D9TNZ0	Alpha-galactosidase	Tthe_0140	83.958	1
D9TR57	Beta-glucosidase	Tthe_1813	51.651	7
P29441	Xylose isomerase	xylA	50.183	21
D9TTQ7	Xylulokinase	Tthe_2491	54.684	5
D9TMP1	Aldehyde–alcohol dehydrogenase	Tthe_2646	94.723	11
D9TP56	Iron-containing alcohol dehydrogenase	Tthe_1156	43.352	11
D9TR64	Iron-containing alcohol dehydrogenase	Tthe_1821	39.632	3
D9TSE3	Iron-containing alcohol dehydrogenase	Tthe_0472	42.453	6
D9TTR6	Iron-containing alcohol dehydrogenase	Tthe_2500	43.298	6

### Identification and characterization of AdhE enzyme from *Thermoanaerobacterium* sp. M5

**Fig. 1:**
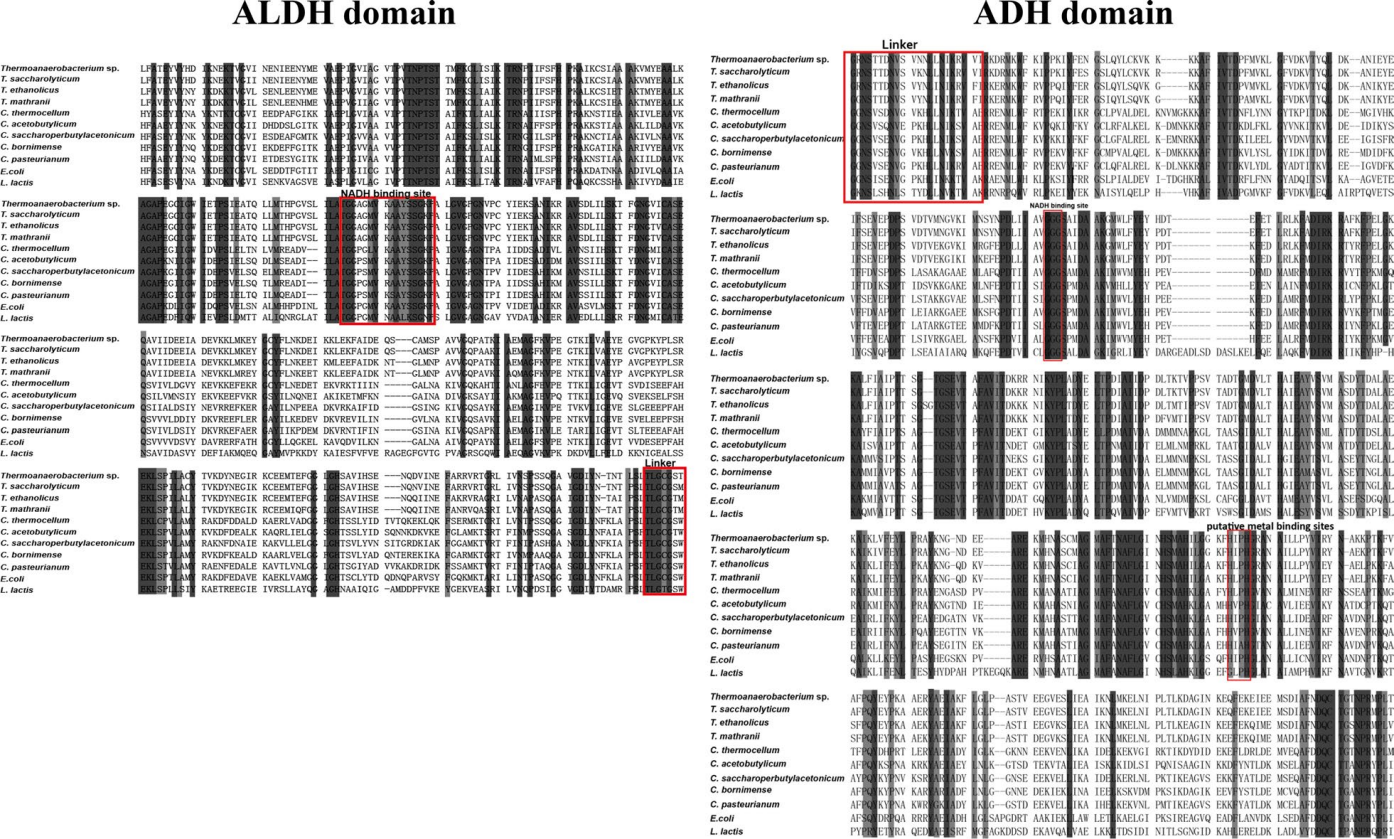
Characterization of butanol-catalyzing and thermostable AdhE enzyme of *Thermoanaerobacterium* sp. M5. The results of blasting various AdhE from different strains. (1) Determination of the optimum pH. Error bars correspond to the standard deviation of three measurements

**Fig. 2:**
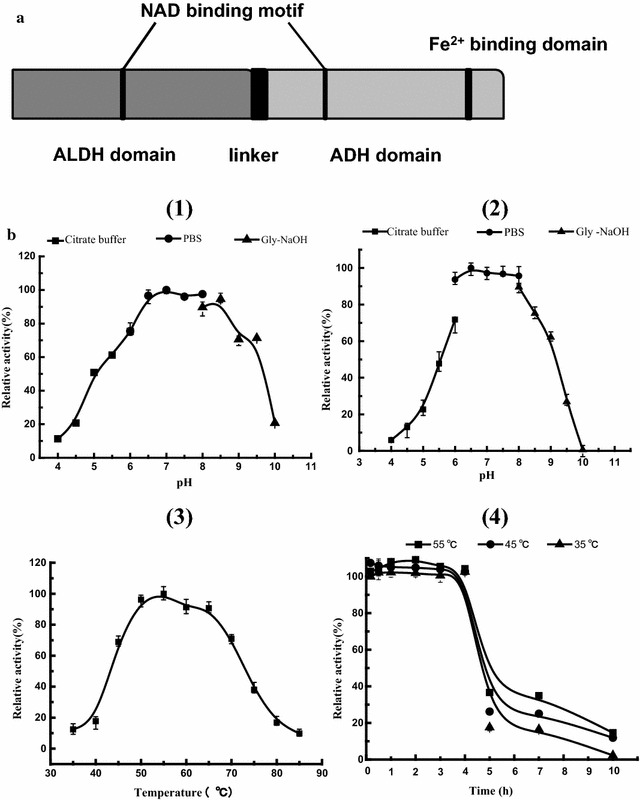
Characterization of butanol-catalyzing and thermostable AdhE enzyme of *Thermoanaerobacterium* sp. M5. **a** The predicted structure of AdhE. **b** Effects of temperature and pH on enzyme activity and stability. Determination of (1) the optimum pH; (2) pH stability; (3) the optimum temperature; (4) thermal stability. Error bars correspond to the standard deviation of three measurements

AdhE is the key enzyme in solventogenic strains responsible for the catalysis of CoA into solvents. Generally, solventogenic clostridia possess two AdhE (AdhE1 and AdhE2) [20]. However, only one *adhE* was annotated in the genome of strain M5 with predicted molecular mass of 94.7 kDa, which was also identified by LC–MS/MS (D9TMP1). Furthermore, no aldehyde dehydrogenase (ALDH) within the annotated CDS of strain M5 was identified, which generally could reduce acetyl-CoA/butyryl-CoA to acetaldehyde/butyraldehyde, proving that *adhE* was the only crucial enzyme for BE production. Actually, deletion of *adhE* in *Thermoanaerobacterium* sp. could lead to the elimination of solvent production [20]. Further bioinformatic analysis showed that AdhE from strain M5 was divided into two domains, ALDH and alcohol dehydrogenase (ADH), which were connected by a conserved linker sequence, indicating it may play biofunction in BE production (Fig. 2a). The ALDH domain is located at the N-terminal, while the ADH domain is located at the C-terminal, which is iron dependent. Both ALDH and ADH possess an NADH binding site, and the two domains are connected by a small conserved linker sequence containing a GXGXXG motif and a putative nucleotide binding region (Fig. 1) [2, 21]. Through the blast of protein data bank archive (pdb) database in the NCBI website, a putative Fe^2+^ binding site is further identified in the ADH domain, suggesting that supplementation of Fe^2+^ may help improve AdhE activity and butanol production.

To better characterize this enzyme, *adhE* was successfully expressed in *E. coli* BL21 (DE3) and purified using Ni^2+^–NTA resin. SDS-PAGE (12%) showed that the molecular weight of AdhE was approximately 95 kDa, which is similar to the predicted 94.7 kDa. Especially, the recombinant AdhE possessed a high BDH activity of 76.31 U/mg, which is higher than 18.07 U/mg of BDH from *C. ljungdahlii* and 0.18 U/mg of AdhE2 from *C. acetobutylicum* [22, 23]. The recombinant AdhE displayed its maximum BDH activity at 55 °C; it also exhibited more than 90% of maximum BDH activity between 50 and 65 °C (Fig. 2b (3)). Thermostability analysis showed that AdhE retained more than 90% of activity at 55 °C for nearly 4 h (Fig. 2b (4)), suggesting that AdhE is thermostable [24]. On the other hand, AdhE exerted high levels of activity at a wide pH range of 6.5–8.5; however, almost no activities were detected at pH below 4.0 or above 9.5 (Fig. 2b (1)). pH stability assays showed that the purified enzyme retained more than 70% of its activity at pH 6.0–8.5 for 20 h, whereas its stability decreased significantly at pH values below pH 6.0 or above pH 9.0 (Fig. 2b (2)). The data shown here could further guide the process optimization for improvement of butanol production.

### Improvement of butanol production through process optimization

pH has been proved to be a key factor in the ABE fermentation process, and generally solventogenic *Clostridium* sp. shows a bi-phase fermentation mode, which includes acidogenic and solventogenic phases. Part of acids formed in the acid phase will be re-assimilated for further solvent production, accompanied by the rebounce of pH values [25]. However, strain M5 did not show the typical bi-phase fermentation mode, in which acids (acetate and butyrate) and solvents (ethanol and butanol) constantly increased along with the fermentation duration. pH optimization experiments showed that slight alkaline environment at pH 7.5 is more suitable for butanol production, which is completely different from the reported acidic environment at pH 4.5–5.5 using solventogenic clostridia (Table 3) [26]. This is consistent with our finding that the optimum pH for BDH activity is between 6.5 and 8.5 (Fig. 2b). 0.60 g/L of butanol was finally obtained from 30 g/L of xylan, which showed almost 100% increase compared to the one without pH control. When strain M5 was cultivated under the pH values below 6.0 or above 9.0, less than 0.10 g/L of butanol occurred.

**Table 3:** Effects of fermentation pH on metabolic product profiles

pH	4.0	5.0	5.5	6.0	6.5	7.0	7.5	8.0	9.0	10.0
Butanol (g/L)	0.020 ± 0.011	0.037 ± 0.015	0.055 ± 0.008	0.097 ± 0.027	0.297 ± 0.058	0.467 ± 0.050	0.596 ± 0.067	0.489 ± 0.087	0.093 ± 0.002	0.046 ± 0.001
Ethanol (g/L)	0.538 ± 0.019	0.602 ± 0.001	0.633 ± 0.064	0.644 ± 0.141	0.976 ± 0.057	1.212 ± 0.460	2.270 ± 0.375	2.438 ± 0.258	1.942 ± 0.426	0.335 ± 0.094
Acetate (g/L)	0.610 ± 0.026	3.980 ± 0.050	4.052 ± 0.065	3.351 ± 0.534	2.947 ± 0.102	3.061 ± 0.763	3.340 ± 0.211	2.909 ± 0.820	2.834 ± 0.356	0.413 ± 0.108
Butyrate (g/L)	0.539 ± 0.134	4.950 ± 0.207	6.041 ± 0.241	5.385 ± 0.623	4.272 ± 0.297	3.095 ± 0.214	3.052 ± 0.548	2.164 ± 0.679	2.533 ± 0.475	0.157 ± 0.389

Error bars correspond to the standard deviation of triplicates

**Fig. 3:**
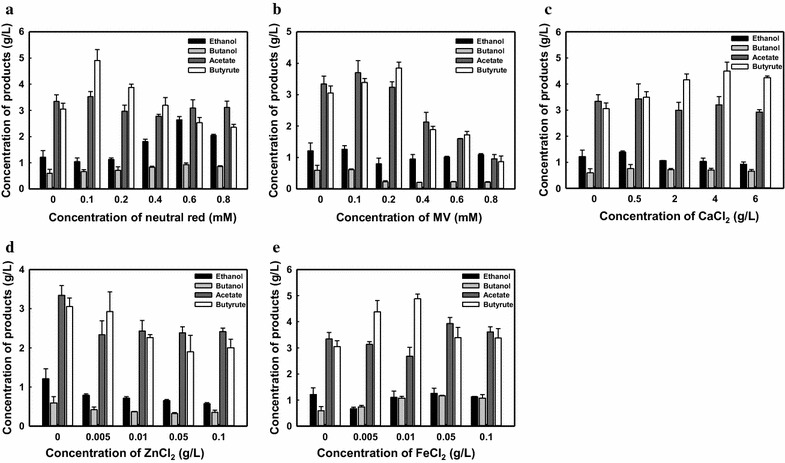
Effects of different AECs and divalent ions on butanol production for *Thermoanaerobacterium* sp. M5. Fermentation profiles of *Thermoanaerobacterium* sp. M5 in medium containing **a** NR, **b** MV, **c** CaCl_2_, **d** ZnCl_2_, **e** FeCl_2_. Error bars correspond to the standard deviation of three measurements

Reducing power has been reported as a limiting factor for reductive chemicals synthesis, such as butanol [27]. Furthermore, BDH was characterized as NAD(P)H dependent. Hence, to further drive metabolic flux toward butanol synthesis, artificial electron carriers (AEC), such as neutral red (NR) and methyl viologen (MV), were supplemented in the fermentation initially. As seen in Fig. 3a, when NR was increased to 0.6 mM, butanol production was significantly improved from 0.60 to 0.93 g/L. Meanwhile, the amount of butyrate was decreased when NR was increased from 0.1 to 0.8 mM, suggesting that the carbon flux may be altered toward butanol production through CoA-transferase. However, the supplementation of MV gave even lower butanol production (Fig. 3b), which may be because MV inhibits bacterial growth [28].

Some divalent ions, such as Fe^2+^, Ca^2+^ and Zn^2+^, also influence butanol production [29–31]. Figure 3c shows that the addition of CaCl_2_ (0.5–6.0 g/L) did not help boost butanol production, and the addition of ZnCl_2_ even lowered butanol production (Fig. 3d). On the contrary, the addition of FeCl_2_ in the culture could obviously enhance butanol production. The highest butanol production of 1.17 g/L was achieved with supplementation of 0.05 g/L FeCl_2_ in the culture, which was almost twofold higher than that of the control groups (Fig. 3e).

### High butanol production by co-cultivation of *Thermoanaerobacterium* sp. M5 and *C. acetobutylicum* NJ4

**Fig. 4:**
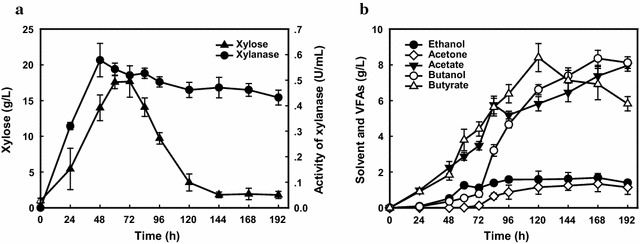
Fermentaion profiles using a co-cultivation system consisting of *Thermoanaerobacterium* sp. M5 and *C. acetobutylicum* NJ4. **a** Time-dependent enzyme activities, xylose concentration and **b** product concentrations in the co-cultivation system consisting of culture *Thermoanaerobacterium* sp. M5 and *C. acetobutylicum* NJ4 from 60 g/L beechwood xylan

*Thermoanaerobacterium* sp. M5 could efficiently degrade up to 90 g/L of xylan; however, the final butanol titer was not improved significantly and a high amount of reducing sugars was still left over in the fermentation broth. Hence, to further improve the final butanol titer, a one step process by co-cultivation of *Thermoanaerobacterium* sp. M5 and *C. acetobutylicum* NJ4 was set up for improvement of butanol production. In this co-cultivation system, *Thermoanaerobacterium* sp. M5 was firstly grown on 60 g/L of beechwood xylan at 55 °C, and 0.58 U/mL xylanase and 7.69 U/mL β-xylosidase were secreted into the culture medium (Fig. 4a). The xylan-degrading enzymes could readily hydrolyze xylan into xylose in the culture medium and low levels of ethanol (1.11 g/L), butanol (0.78 g/L), acetic acid (3.47 g/L) and butyric acid (4.41 g/L) were synthesized within 72 h (Fig. 4b). Subsequently, the solventogenic *C. acetob*utylicum NJ4 isolated by our laboratory was further inoculated into *Thermoanaerobacterium* sp. M5 culture medium. As seen in Fig. 4a, xylanase and β-xylosidase activities were still relatively stable in the culture medium after the incubation of strain NJ4, indicating that active enzymes still performed hydrolytic functions for continuous hydrolysis of xylan to fermentable reducing sugars. More importantly, the addition of *C. acetobutylicum* NJ4 to the *Thermoanaerobacterium* sp. M5 culture boosted utilization of xylose accumulated in the medium and gave a high 1.68 g/L of ethanol and 8.34 g/L of butanol, corresponding to a butanol and solvent yield of 0.14 and 0.17 g/g, respectively. The high initial xylose concentration (17.6 g/L), constantly stable xylanase activity (~ 0.5 U/mL) and high butyric acid concentration (3.8 g/L) may contribute to butanol formation after inoculation of culture NJ4. Results in this study demonstrated the potential of converting lignocellulosic substrates directly to fuels by using this co-cultivation system, consisting of thermophilic strain M5 which efficiently releases xylose from xylan for further biofuel and biochemicals synthesis.

## Discussion

In this study, the newly isolated thermophilic *Thermoanaerobacterium* sp. M5 shows capability of both xylan degradation and butanol biosynthesis. This represents the first report of biosynthesis of butanol from xylan via CBP under thermophilic conditions, which is supported by the following: (i) high xylanase (0.26 U/mg) and xylosidase (3.81 U/mg) activities could be detected when using xylan as the sole carbon source; (ii) key enzymes conducting xylan degradation (mainly xylanase and xylosidase) and butanol formation (mainly AdhE) were indentified by LC–MS/MS; (iii) 1.17 g/L of butanol was obtained from 30 g/L of xylan through CBP, and the highest 8.34 g/L of butanol occurred from 60 g/L of xylan by co-cultivation with solventogenic *C. acetobutylicum* NJ4, which was the highest butanol production from xylan through CBP (Table 4) [32]. Moreover, the acetone-uncoupled production within *Thermoanaerobacterium* sp. M5 suggests that the acetone metabolic pathway may be deficient or blocked. Indeed, the acetoacetate decarboxylase (*adc*) gene catalyzing the conversion of acetoacetate into acetone was eliminated in the genome of strain M5, which is the last step in the acetone formation pathway. This acetone-uncoupled BE fermentation pathway could further reduce the subsequent cost of separation. These metabolic properties of strain M5 could greatly improve the economic viability of biobutanol production both in terms of the associated substrate costs, by-products and the downstream separation complexities.

**Table 4:** Comparision of butanol production by wild-type and genetically engineered thermophilic bacteria

Strain	Description	Substrate	Titer (g/L)	References
*T. saccharolyticum* JW/SL-YS485	Wild type	Xylose	0	[12]
*T. saccharolyticum* JW/SL-YS485	Integration of butanol synthetic way in the wild type	Xylose	0.85	[12]
*T. saccharolyticum* JW/SL-YS485	Integration of butanol synthetic way in the lactate-deficient strain	Xylose	1.05	[12]
*T. thermosaccharolyticum* DSM 571	Overexpression of the butyryl-CoA formation (*thl, hbd, crt, bcd, etfA, and etfB*)	Xylose	0.38	[20]
*T. thermosaccharolyticum* W16	Wild type	Corn stover	0.074	[11]
*Thermoanaerobacterium* sp. M5	Wild type	Xylan	1.17 g/L	This study

**Fig. 5:**
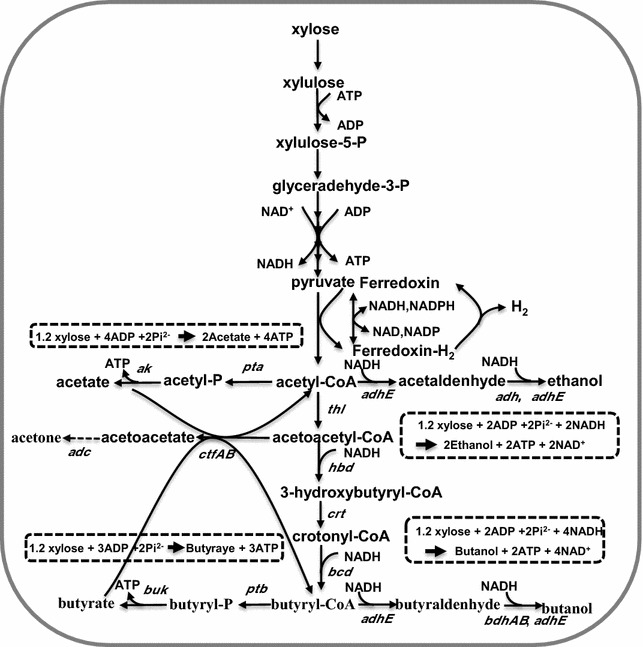
The proposed metabolic pathway for butanol–ethanol (BE) production within *Thermoanaerobacterium* sp. M5. *ak* acetate kinase, *pta* phosphotrans acetylase, *adhE* acetaldehyde dehydrogenase, *adh* alcohol dehydrogenase, *thl* thiolase, *ctfAB* CoA-transferase, *hbd* 3-hydroxybutyryl-CoA dehydrogenase, *crt* crotonase, *bcd* butyryl-CoA dehydrogenase, *bdh* butanol dehydrogenase, *ptb* phosphotransbutyrylase, *buk* butyrate kinase

Biobutanol was generally produced by solventogenic *Clostridium* sp. in the traditional ABE fermentation with the mass ratio of 3:6:1. However, it was found that strain M5 produced higher ethanol over butanol. For example, under optimized pH conditions, 0.60 g/L of butanol occurred with 2.27 g/L of ethanol (Table 3). It is known that the reducing power is a limiting factor for butanol production, as 4 mol NAD(P)H will be required for 1 mol butanol production with 2 mol NAD(P)H generation from 1 mol glucose or 1.2 mol xylose (Fig. 5). Generally, electron carriers could inhibit hydrogenase in the electron transport shuttle system to overproduce NADH; hence, supplementation of exogenous electrons carriers could improve the NAD(P)H pool size in vivo, thus further driving toward butanol production [33]. Comprehensive studies have been carried out to improve butanol production by using this method [34–36]. For example, Jiang et al. have produced 6.09 g/L butanol with 20.89% increased yield through extra addition of 0.79 g/L NR [37]. In most of the studies, MV replaces the functions of ferredoxin as a better substrate for ferredoxin–NAD(P)^+^ reductase, creating an artificial electron transport chain [38]. However, in strain M5, MV, also known as a commercial herbicide “paraquat”, did not help improve butanol production due to a certain inhibition of bacterial growth. Instead, NR shows lower inhibition compared to MV and could significantly improve butanol production. On the other hand, butanol production was also improved notably with addition of Fe^2+^, because ferredoxin could be properly synthesized due to iron existence, which indicates that Fe^2+^ could enhance butanol production maybe because of NADH level improvement.

CBP has been considered to be the ideal solution for cost-effective hydrolysis and fermentation of lignocellulosic biomass into biofuels [39]. Unlike most of the solventogenic *Clostridium* species, the strain M5 shows capability of direct butanol and ethanol production under thermophilic conditions from polysaccharides, such as xylan, representing another reported wild-type solventogenic species which could directly convert xylan to butanol (Table 4). The success of butanol production via CBP was attributed to both its efficient secretion of xylan-degrading enzymes, such as xylanase and β**-**xylosidase, and the existence of the butanol synthetic pathway (Fig. 5). Actually, through overexpression of genes responsible for butanol formation (*thl*, *hbd*, *crt*, *bcd*, *etfA*, and *etfB*) in wild-type thermophilic *T. thermosaccharolyticum* DSM571, 0.38 g/L of butanol was obtained from xylose [20]. In our study, a higher 1.17 g/L of butanol was produced from xylan after fermentation optimization by strain M5 compared to the previous study; however, butanol production was still much lower than that using monosubstrates as the carbon sources by *Clostridium* strains. Instead, more ethanol and VFAs were produced, especially using polysaccharides as the substrate. The reason could be due to the weak driven carbon flux toward butanol formation. Similar results were also obtained from cellulose when using genetically engineered cellulolytic strains. For example, metabolic construct of cellulolytic *C. cellulovorans* after introduction of the *adhE2* gene could only produce a low amount of butanol (1.6 g/L) from cellulose [40]. Nevertheless, a high xylose production of 17.6 g/L, and stable xylanase (0.58 U/mL) and β-xylosidase (7.69 U/mL) activities still occurred when using 60 g/L of xylan as the substrate by strain M5. Based on this characteristics, setting up a co-cultivation system with another solventogenic strain is an efficient and simple strategy to further promote butanol production. Indeed, *C. acetobutylicum* NJ4 could further accelerate xylose utilization and drive carbon flux toward butanol production. The final butanol production was improved to 8.34 g/L, which is higher than the highest report of 7.9 g/L butanol from cellulose using co-cultivation systems containing *C. thermocellum* and *C. saccharoperbutylacetonicum* N1-4 [41]. It should be noticed that butyric acid generated before the inoculation of strain NJ4 could also trigger butanol production, as the intracellular and/or extracellular levels of butyric acid are implicated in the induction of solventogenesis in *C. acetobutylicum* [42]. However, further studies are still needed to improve the final titer through overexpression of *adhE* in vivo in strain M5 and elimination of the by-products pathway simultaneously.

## Conclusion

The newly isolated *Thermoanaerobacterium* sp. M5 could be regarded as a potential candidate for butanol production from xylan via the CBP process under thermophilic conditions without detectable acetone. The wild-type strain M5 possesses the capacity of efficient xylan utilization through secretion of xylanases and β-xylosidases, and 1.17 g/L of butanol was also obtained from xylan after optimizing the fermentation conditions based on the characteristics of AhdE. The characterized thermostable butanol-producing key enzyme, AdhE in strain M5, is the sole enzyme responsible for butanol formation, which has the activity of aldehyde dehydrogenase in vitro. In addition, due to the limiting capacity of butanol production for strain M5, the co-cultivation system consisting of *Thermoanaerobacterium* sp. M5 and *C. acetobutylicum* NJ4 was established and the final butanol production was significantly improved to 8.34 g/L. The results of this study offer fundamental knowledge for further improvement of butanol production in thermophiles, and the limited factors of butanol-producing metabolic pathway for strain M5 should also be further studied.

## Abbreviations

CBP: consolidated bioprocessing
AdhE: bifunctional alcohol/aldehyde dehydrogenase
BE: butanol–ethanol
ABE: acetone–butanol–ethanol
BDH: butanol dehydrogenase
CBMs: carbohydrate-binding modules
ADH: alcohol dehydrogenase
ALDH: aldehyde dehydrogenase
AEC: artificial electron carriers
NR: neutral red
MV: methyl viologen
